# Number of Remaining Teeth and Its Association with Educational Level in Chilean Adults: Data from the National Health Survey 2016-2017

**DOI:** 10.1155/2020/8848190

**Published:** 2020-08-31

**Authors:** Paula Margozzini, Rodrigo Berrios, Rosario García-Huidobro, Claudia Véliz, Carolina del Valle, Juan Pablo Vargas, Oslando Padilla, Duniel Ortuño

**Affiliations:** ^1^Departamento de Salud Pública, Facultad de Medicina, Pontificia Universidad Católica de Chile, Santiago, Chile; ^2^Escuela de Odontología, Facultad de Medicina, Pontificia Universidad Católica de Chile, Santiago, Chile; ^3^Ministerio de Salud de Chile, Santiago, Chile

## Abstract

**Introduction:**

Several population studies have addressed oral health inequalities. Edentulism, functional dentition, and number of remaining teeth have been associated with different socioeconomic level measurements. The aim of this study was to evaluate the association between educational level and tooth loss in the Chilean population aged 15 years and above, based on the 2016-2017 National Health Survey (ENS 2016-2017). *Material and Methods*. The sample for this cross-sectional study comprised 5473 subjects. The main independent variable was educational level (LEL: low, MEL: medium, and HEL: high). To measure tooth loss, we considered the variables number of remaining teeth, edentulism, and functional dentition. We used logistic regressions to assess the condition of dentition according to the subject's EL. As to the number of teeth variable, linear regressions were conducted. The analyses were carried out considering the complex sampling design in SPSS 24.0.

**Results:**

When comparing LEL subjects with HEL subjects, the adjusted difference in number of remaining teeth was 3.11 for the maxilla and 1.72 for the mandible. An individual with LEL had a 7.51 [3.50–16.10] and 6.06 [2.68–13.68] times higher risk of upper edentulism and lower edentulism than a HEL individual, respectively. Regarding functional dentition, the adjusted OR in HEL subjects was 13.33 [8.02–22.15] and in MEL subjects was 2.81 [2.03–3.87], compared to LEL results.

**Conclusions:**

LEL was associated with a significant tooth loss in the Chilean population. Subjects with LEL obtained a lower mean of number of remaining teeth and higher prevalence of edentulism and nonfunctional dentition.

## 1. Introduction

Tooth loss is the main cause of the burden of disease due to oral conditions in the world [1] and has been related to coronary artery disease [2], metabolic syndrome [3], and diabetes [4]. The consequences of tooth loss depend on the severity and intraoral location of the lost teeth [5]. Also, a lower number of teeth reduces the capacity for social interaction and the quality of life [6].

A variety of parameters are used to measure tooth loss, including edentulism, functional dentition, and number of remaining teeth [7]. Edentulism is considered “the dental equivalent of mortality” [8]. Also, this condition is an independent factor in predicting general mortality [9]. The prevalence of edentulism is an independent indicator of oral health at the population level [10]. Functional dentition is defined as the presence of at least twenty permanent teeth in an individual [11].

In the last two decades, a decrease in the prevalence of severe tooth loss (having between 1 and 9 remaining teeth) has been reported [12]. However, in the USA and other countries, demographic growth and population aging will affect the slow reduction of the prevalence of edentulism [13]. In Brazil, the rates of edentulism have continued almost unchanged between 2003 and 2010, whereas nonfunctional dentition is present in about a quarter of the older population [14]. According to the first National Health Survey (ENS 2003) in Chile, the prevalence of edentulism was 5.5% [4.6–6.4], where women showed a higher tooth loss. Only 20% of the adult population between 35 and 44 years in Chile still has a complete denture, whereas, in those subjects aged from 65 to 74 years, the prevalence was 1% [15]. In Chilean women between 45 and 59 years, edentulism is the third specific cause of disease burden, being 2.8 times higher than in men [16].

Several population studies have addressed oral health inequity-related issues, but those are scarce in Chile. Number of remaining teeth, edentulism, and functional dentition have been associated with different socioeconomic level measurements [17, 18], including the educational level (EL), which is considered a key factor for health status [19]. As to number of teeth, relevant socioeconomic gradients have been found in the United States [20] and other countries [21].

According to Elani et al. [5], Chile had a higher educational level (EL) gradient in terms of functional dentition and number of teeth compared with Australia, Canada, New Zealand, and the United States. The previous study used data of the Chilean population from the national survey performed in 2003 (ENS 2003) [15]; therefore, a new analysis is necessary in Chile, considering the recent publication of the results from the National Health Survey (ENS 2016-2017). The aim of this study was to analyze the association between EL and tooth loss of the Chilean population, aged 15 years and above, based on the ENS 2016-2017 data.

## 2. Materials and Methods

This study worked on the data obtained from the ENS 2016-2017, which was conducted by the Ministry of Health of Chile. The ENS 2016-2017 is a tool for national epidemiological surveillance that focuses on non-communicable diseases. This survey is a cross-sectional design, consisting of a complex, random, stratified, and multistage cluster oversampling representative of Chilean national, regional, and location levels. The ENS 2016-2017 assessed 72 health problems and their social and biological determinants.

Data on all subjects recruited for the oral examination stage in the ENS 2016-2017 were used for this study, having a final sample size of 5473 subjects (missing data 47/5520). It included people aged 15 years and above, who were permanent residents of the selected households and had undergone a complete dental examination. Pregnant women and people with violent behavior during on-site visits were excluded from the survey.

Trained and calibrated nurses performed the dental examination in their second home visit to the respondents, where the presence of cavitated caries, use of a prosthesis, and the number of teeth were measured. A dental mirror, dental explorer, and standard operation lamp were used. According to the pilot study ENS 2003 (*n* = 105), sensitivity level to detect missing teeth and dental fillings was 70% [15] and interexaminer reliability was substantial (kappa = 0.75, *p* value <0.01) [22].

In the ENS 2016-2017, nurses were trained by nine dentists, through a demonstration, a dental examination practice, and a final test with twenty clinical cases. The test average score was 49.95 (±2.74) and interexaminer reliability was substantial (kappa = 0.85, *p* value <0.01) [22].

The analysis was carried out based on the ENS 2016-2017 data, available at http://epi.minsal.cl/encuestas-poblacionales. The estimations were performed using the complex sampling module of the SPSS program, version 24.0 (Mac OS X) (SPSS Inc., Chicago, IL, USA).

The main independent variable of this study was EL. Based on the data obtained from the household socioeconomic module in the ENS 2016-2017, values for the highest EL or current EL and subjects' last approved course were known. We used this information to establish the total number of years of study, variable that was then categorized into LEL (low educational level = less than eight years), MEL (medium educational level = between 8 and 12 years), and HEL (high educational level = more than 12 years).

The three dependent variables were number of remaining teeth, edentulism, and functional dentition. For the variables edentulism and functional dentition, the adjusted prevalence with their respective 95% confidence intervals (CI 95%) were estimated, while for the variable number of remaining teeth, the means were obtained with the respective standard errors.

Concerning the statistical analysis, we evaluated the association between EL and edentulism or functional dentition by logistic regressions. These models provided adjusted odds ratios (OR) with CI 95%. Also, multiple linear models were performed to compare the mean number of remaining teeth by EL. For all the adjusted comparisons, we included the covariates sex (men/women), age (groups: 15–24, 25–44, 45–64, and 65 and above), and location (rural or urban). For sex comparisons, we used linear regression models when the number of remaining teeth was evaluated and logistic regression models when edentulism or functional dentition was considered. The significance level was fixed at 0.05.

The Scientific Ethics Committee of the Faculty of Medicine of Pontificia Universidad Católica de Chile (CEC-MEDUC) approved the protocols and informed consents of this study nested in the ENS 2016-2017 (project number: 16-019). Also, we obtained written consent from all the cases. For participants under 16 years of age, written informed consent was obtained from their parent or guardian.

## 3. Results

A total of 5473 subjects were included in the study, reporting a mean of 43.13 years, of which 63.4% were female. The respondent distribution by EL was 24.3% for LEL, 53.9% for MEL, and 21.8% for HEL (Table 1).

### 3.1. Number of Remaining Teeth

The adjusted mean number of teeth in the maxilla was 10.88 [10.68–11.10] and in the mandible was 11.86 [11.51–11.86]. In men, the mean number of upper teeth was 10.97 [10.65–11.28] and the mean number of lower teeth was 12.0 [11.73–12.27]. In women, the mean number of upper teeth was 10.29 [9.99–10.58] and the mean number of lower teeth was 11.35[11.09–11.6].

The adjusted difference in the number of teeth in the maxilla was 1.59 when comparing LEL with MEL subjects (*p* value <0.001), whereas this difference increases up to 3.11 (*p* value <0.001) between LEL and HEL subjects. A comparison between MEL and HEL subjects resulted in a difference of 1.52 teeth in the maxilla (*p* value <0.001) (Table 2).

For the mandible, the adjusted difference was 0.83 when comparing LEL and MEL subjects (*p* value <0.001). Between LEL and HEL subjects, the difference was 1.72 teeth (*p* value <0.001). When comparing MEL and HEL subjects, the mean difference obtained was 0.89 teeth in the mandible (*p* value <0.001). In men with a HEL, the adjusted mean of upper teeth was 12.51 [12.14–12.88], whereas in those with an LEL, it was 9.40 [8.76–10.05]. In women with a HEL, the mean of upper teeth was 11.83 [11.46–12.20], but those with an LEL decreased to 8.72 [8.08–9.36]. In men with a HEL, the mean of lower teeth was 12.87 [12.59–13.15], and in those with an LEL, it was 11.15 [10.60–11.70]. In women, the difference in lower teeth between HEL with LEL (*p* value <0.001) amounted to 1.72. When comparing the number of remaining teeth between men and women, according to EL, significant differences were found in both the maxilla and mandible (*p* value <0.001) (Table 2).

### 3.2. Edentulism

The adjusted prevalence of edentulism in the maxilla was 8.92% [8.25–9.59%], whereas in the mandible, it was 5.36% [4.91–5.81%]. The prevalence of upper edentulism was 9.60% [8.82–10.38%] in men and 16.56% [15.58–17.54%] in women. As to the mandible, the prevalence of edentulism was 5.67% [5.06–6.28%] and 9.84% [9.05–10.63%] in men and women, respectively. Women showed an edentulism OR of 2.42 [1.71–3.42] for the maxilla and 2.30 [1.50–3.53] for the mandible in relation to men.

An MEL individual had a 3.36 [1.50–7.52] times higher risk of edentulism than a HEL individual, while an LEL individual had a 7.51 [3.50–16.10] times higher risk of having no teeth than a HEL individual (Table 3). As to the mandible, a MEL individual had a 3.07 [1.33–7.09] times higher risk of edentulism than a HEL individual. Moreover, the same risk increases to 6.06 [2.68–13.68] in an LEL subject compared to a HEL individual (Table 4).

In men with LEL, the prevalence of upper edentulism was 23.41% [19.56–27.26], whereas in those with a MEL and HEL, it was 3.8% [3.34–4.43] and 1.50% [0.09–2.08], respectively. In women, the prevalence of upper edentulism was 42.35% [38.71–45.99] for LEL, 6.30% [5.55–7.05] for MEL, and 1.02% [0.07–1.30] for HEL. All differences in the prevalence described above were statistically significant (*p* value <0.001) (Table 3).

The prevalence of lower edentulism was 15.12% [12.31–17.94] in LEL, 1.83% [1.56–2.10] in MEL, and 0.06% [0.03–0.08] in HEL. The prevalence of lower edentulism in women was 25.40% [22.82–27.9] for LEL, 4.03% [3.53–4.5] for MEL, and 0.09% [0.07–1.18] for HEL. All differences were statistically significant after the adjustments (*p* value <0.001) (Table 4).

### 3.3. Functional Dentition

The adjusted prevalence of functional dentition was 75.30% [73.98–76.62], and the OR between men and women was 1.50 [1.14–1.96]. In terms of EL, the prevalence was 28.82% [25.51–32.13] for LEL, 79.53% [78.18–80.88] for MEL, and 94.42% [93.23–95.61] for HEL. Comparing LEL subjects with MEL and HEL subjects, the obtained OR were 2.81 [2.03–3.87] and 13.33 [8.02–22.15], respectively. In men, the prevalence of functional dentition was 31.83% [26.04–37.61] for LEL, 82.20% [80.36–84.04] for MEL, and 94.50% [92.83–96.15] for HEL. In women, the prevalence of functional dentition was 26.74% [22.86–30.62] for LEL and amounted to 94.34% [93.06–95.63] in those with a HEL (Table 5).

## 4. Discussion

This study aimed to assess the effect of EL on tooth loss based on the ENS 2016-2017 data. The results confirmed the hypothesis that in Chile remains a robust educational gradient, where individuals with an LEL have the worst levels of edentulism, number of teeth, and functional dentition. This tendency was observed in men and women, reporting a higher difference in the maxilla. Our findings conform with results performed in other populations where an increase in EL was inversely associated with tooth loss [23, 24]. Matsuyama et al. suggested a causal effect of education in the size of 9% points decrease in the probability of edentulism per additional year of education in a Britain cohort [24]. Kim et al. found that as income and education levels increased, subjects were more likely to have 20 remaining teeth after adjusting for similar covariates of our study (*p* value and *p* value for trend <0.001) [18].

Subjects with a HEL showed better conditions in terms of number of teeth, which is consistent with that a HEL relates to higher self-control of health behaviors [25]. Knowledge and skills acquired through years of study have a positive influence on how people consider educational information and access to health services [26]. In this study, education was measured as a categorical variable, thereby acknowledging the relevance of educational achievements, which would explain the better oral health levels [27]. One limitation of our study was that this variable does not measure the quality of educational experiences, which is quite essential to specify the role of education in health outcomes.

Other studies have concluded that inequities in oral health are not limited to individual factors, such as treatment adherence or self-care behaviors [28]. Preventive interventions based on an individual approach have not been sufficient to reduce inequities relating to tooth loss [29]. On the other hand, preventive interventions that focus on population aim at addressing disease causes, changing determinants concerning environmental, psychosocial, economic, and political aspects [30]. A strategical combination of individual and population preventive approaches is necessary to reduce inequities in oral health in Chile and the world [31].

According to the ENS 2003, the prevalence of edentulism for LEL subjects was 10.30%, whereas that for MEL and HEL individuals was 1.60% and 0.30%, respectively. Our findings indicated that inequities have persisted despite the implementation of new oral health programs in the country. Although the national prevalence of nonfunctional dentition decreased from 32.30% to 24.70% between 2003 and 2017, the differences in oral health outcomes, according to EL, have increased. This situation of substantial inequities in Chile presents the challenge of establishing a more focused approach to the social determinants of oral health and not implementing programs with only individual prevention strategies.

On the other hand, dental extraction continues to be a common alternative of dental care in the Chilean oral health programs. It is the same reality that can be found in other countries, where despite the reduction of the prevalence and severity of caries, excessive indications for dental extractions lead to a lower number of teeth [32]. Also, the educational items of oral health programs impact differently on the varied social groups, increasing inequities in oral health [33].

The observed educational gradient is also visible in other chronic conditions, such as hypertension, dyslipidemia, and obesity, among others, in Chile [34]. The mean higher age of people with LEL could partially explain the social gradient in dental outcomes. However, our analyses showed significant gradients, even after adjusting for age. It is necessary to study other factors that determine that the socially more vulnerable strata have a higher burden of morbidity in Chile. Moreover, there is a need to implement comprehensive public health policies that focus on social determinants shared with other chronic diseases and consider oral health an integral part of general health [35]. Our findings suggest that using one policy instrument, that is raising the level of education, governments can achieve two aims simultaneously: raise the wealth of the population and improve health, including oral health [36]. In countries like most western European countries, the remarkable increase in the education level during the second half of the last century was associated with better health [36]. New studies are also required to analyze the impact and efficiency of educational policies on oral health indicators like number of remaining teeth.

The main limitation of this study was the impossibility of establishing causation. Education, unlike occupation or income, is a stable indicator of socioeconomic position, less susceptible to the reverse causation phenomenon. Another limitation was that other indicators of socioeconomic position were not included. The use of different indicators of socioeconomic position probably would result in gradients with similar slopes. A third limitation was that although the participants were classified into three groups of EL, the specific effect, according to the birth cohort, was not established. This fact may produce a bias of over representation of subjects that belong to higher age cohorts with LEL [37]. Finally, we recognized that analysis of population data would never provide a perfect explanation of oral inequalities [38].

## 5. Conclusions

This study showed that LEL was associated with an increased burden of tooth loss in the Chilean population. Subjects with an LEL had a lower mean number of remaining teeth, higher prevalence of edentulism, and lower prevalence of functional dentition, independent of age. The educational gradient relating to oral health remains in Chilean individuals; therefore, it is necessary to reorient public health policies for arrest inequities.

## Figures and Tables

**Table 1:** Survey respondents' general characteristics (mean age: 43.13 y). ENS 2016-2017, Chile.

Variables	*n* (%)
Gender	
Men	2003 (36.6)
Women	3470 (63.4)
Total	5473

Age (years)	
15–24 y	728 (13.3)
25–44 y	1561 (28.5)
45–64 y	1836 (33.5)
65 y and above	1348 (24.7)

EL	
Low (LEL)	1329 (24.3)
Medium (MEL)	2948 (53.9)
High (HEL)	1196 (21.8)

EL, educational level; *n*, sample size.

**Table 2:** Number of teeth in the maxilla and mandible according to EL, *n* = 5473. ENS 2016-2017, Chile.

	Number of teeth in the maxilla			Number of teeth in the mandible		
EL	Mean	Error	CI 95%	Mean	Error	CI 95%
*Low*	9.06	0.321	[8.43–9.69]	10.82	0.271	[10.29–11.36]
M (*n* = 414)	9.40	0.330	[8.76–10.05]	11.15	0.280	[10.60–11.70]
W (*n* = 915)	8.72	0.325	[8.08–9.36]	10.50	0.275	[9.96–11.04]

*Medium*	10.65	0.134	[10.38–10.91]	11.65	0.115	[11.43–11.88]
M (*n* = 1106)	10.99	0.159	[10.68–11.30]	11.98	0.133	[11.71–12.24]
W (*n* = 1842)	10.30	0.137	[10.04–10.57]	11.33	0.124	[11.09–11.57]

*High*	12.17	0.178	[11.82–12.52]	12.54	0.130	[12.29–12.80]
M (*n* = 483)	12.51	0.189	[12.14–12.88]	12.87	0.142	[12.59–13.15]
W (*n* = 713)	11.83	0.190	[11.46–12.20]	12.22	0.143	[11.94–12.50]

Adjusted linear regression models by age, sex, and location. M, men, W, women, and EL, educational level.

**Table 3:** Prevalence of edentulism in the maxilla according to EL, *n* = 5473, ENS 2016-2017, Chile.

EL	Prevalence	CI 95%	OR	CI 95%
*Low*	32.88	[31.63–34.12]	7.51	[3.50–16.10]
M (*n* = 414)	23.41	[19.56–27.26]	—	—
W (*n* = 915)	42.35	[38.71–45.99]	—	—

*Medium*	5.13	[4.63–5.64]	3.36	[1.50–7.52]
M (*n* = 1106)	3.88	[3.34–4.43]	—	—
W (*n* = 1842)	6.30	[5.55–7.05]	—	—

*High*	1.29	[0.01–1.65]	1	—
M (*n* = 483)	1.50	[0.09–2.08]	—	—
W (*n* = 713)	1.02	[0.07–1.30]	—	—

Adjusted logistic regression models by age, sex, and location. M, men, W, women, and EL, educational level.

**Table 4:** Prevalence of edentulism in the mandible according to EL, *n* = 5473. ENS 2016-2017, Chile.

EL	Prevalence	CI 95%	OR	CI 95%
*Low*	21.19	[19.24–23.14]	6.06	[2.68–13.68]
M (*n* = 414)	15.12	[12.31–17.94]	—	—
W (*n* = 915)	25.40	[22.82–27.98]	—	—

*Medium*	2.97	[2.65–3.28]	3.07	[1.33–7.09]
M (*n* = 1106)	1.83	[1.56–2.10]	——	—
W (*n* = 1842)	4.03	[3.53–4.53]	—	—

*High*	0.07	[0.05–0.09]	1	—
M (*n* = 483)	0.06	[0.03–0.08]	——	—
W (*n* = 713)	0.09	[0.07–1.18]	—	—

Adjusted logistic regression models by age, sex, and location. M, men, W, women, and EL, educational level.

**Table 5:** Prevalence of functional dentition according to EL, *n* = 5473. ENS 2016-2017, Chile.

EL	Prevalence	CI 95%	OR	CI 95%
*Low*	28.82	[25.51–32.13]	1	—
M (*n* = 414)	31.83	[26.04–37.61]	—	——
W (*n* = 915)	26.74	[22.86–30.62]	—	—

*Medium*	79.53	[78.18–80.88]	2.81	[2.03–3.87]
M (*n* = 1106)	82.20	[80.36–84.04]	—	—
W (*n* = 1842)	77.03	[75.20–78.86]	—	——

*High*	94.42	[93.23–95.61]	13.33	[8.02–22.15]
M (*n* = 483)	94.50	[92.83–96.15]	—	—
W (*n* = 713)	94.34	[93.06–95.63]	——	—

Adjusted logistic regression models by age, sex, and location. M, men, W, women, and EL, educational level.

## Data Availability

The analysis was carried out based on the ENS 2016-2017 data, available at http://epi.minsal.cl/encuestas-poblacionales/
